# Safety and Effectiveness of a Fixed-Dose Combination of Trypsin, Bromelain, and Rutoside in Wound Management: A Randomized Clinical Trial

**DOI:** 10.7759/cureus.82093

**Published:** 2025-04-11

**Authors:** Bhupesh Dewan, Siddheshwar Shinde, Nisha Motwani

**Affiliations:** 1 Medical Services, Zuventus Healthcare Limited, Mumbai, IND

**Keywords:** bromelain, fixed-dose combination, rutin, systemic oral enzymes, trypsin, wound care

## Abstract

Background

Wound healing following tissue injury is a precisely programmed biological process, typically occurring in a proper sequence and timeframe. While healing is perceived as inevitable, it heavily depends on tissue perfusion, which ensures oxygen and nutrient delivery, removal of waste, and promotion of cellular repair mechanisms. However, complications such as vascular occlusion due to fibrin or cellular debris, microbial contamination leading to infection, ischemic tissue necrosis, and dysregulated inflammation can impair healing. Proper care and repair are crucial to maintaining tissue perfusion and supporting the normal wound healing phases: reaction, regeneration, and remodeling. The use of oral enzymatic agents like trypsin, bromelain, and rutin offers a faster and more reliable method to support wound healing by reducing inflammation and enhancing tissue regeneration. This study was designed to assess the safety and effectiveness of treatment with Tibrolin^®^ (a fixed-dose combination of trypsin 48 mg, bromelain 90 mg, and rutoside 100 mg tablet) in improving wound healing and alleviating acute pain in patients following uncontaminated surgeries.

Methods

A phase-IV, open-label, prospective, multi-center clinical study was conducted on 200 patients after elective, clean, uncontaminated surgery. Patients were randomized (1:1) to receive either Tibrolin^®^ or Chymoral Forte^®^ (trypsin-chymotrypsin) tablets, administered as two tablets to be taken orally thrice a day for seven days postoperatively. The primary outcome measure was the percentage of patients reporting incidences of adverse events, while secondary outcome measures were the mean change in the individual and total score of surgical wounds and the Numerical Pain Rating Scale (NPRS) score from baseline (day 0) to day 7.

Results

Tibrolin^®^ was well-tolerated in the study, with no observed adverse or treatment-related adverse events. At the end of the treatment regimen, all wound healing parameters, including erythema, edema, discharge, induration, local irritation, and tenderness, showed a highly significant improvement (p < 0.001) in both treatment groups. Additionally, there was more than an 85% reduction in NPRS scores reported in both groups. A non-significant difference between groups in pain reduction (p = 0.737) and wound healing symptoms (p = 0.554) confirms that Tibrolin^®^ is as efficacious as trypsin-chymotrypsin when evaluated on days 3, 5, and 7 of treatment. Tibrolin^®^ tablets were regarded as good to excellent for treating wound symptoms by 87% of patients and 94% of investigators.

Conclusion

Our results suggest that Tibrolin^®^ administration favors wound healing and improves wound symptoms, such as edema, inflammation, and pain in the management of elective clean, uncontaminated surgical wounds.

## Introduction

Wound healing is a delicate and utterly complex process, comprising a cascade of interlocking biological events, i.e., hemostasis, inflammation, proliferation, and maturation [1]. Tissue perfusion plays a critical role in this process, as adequate blood flow is essential for delivering oxygen and nutrients necessary for cellular activities involved in healing [2]. However, the presence of impediments within the wound, such as exudate, bacteria, and necrotic or cellular debris, may significantly hinder the healing process [3].

Exudate, the fluid produced by wounds during the inflammatory response, is not merely a byproduct but a vital component of the healing process, essential for tissue repair and regeneration [4]. This fluid contains endogenous proteases, including serine proteinase, cysteine proteinase, aspartic proteinase, and matrix metalloproteinases (MMPs). These proteases prepare the wound bed by degrading extracellular matrix components, such as collagen and elastin, facilitating wound closure and remodeling [5]. Endogenous proteases, delivered to the wound bed via exudate, act as active agents in modulating the wound environment. Membrane-bound proteases also contribute by enabling keratinocytes and endothelial cells to forge a trail as they migrate through tissue [6].

The activity of these degrading enzymes is controlled by tissue inhibitors of metalloproteinases (TIMPs), which not only inhibit MMP activity but also promote cell growth [7]. This equilibrium between proteases and their inhibitors is disturbed in impaired wound healing. As a consequence, factors crucial for wound healing, such as the major proteinase inhibitors (e.g., α1-proteinase inhibitor and α2-macroglobulin), components of the provisional wound matrix (e.g., fibronectin and vitronectin), and growth factors (e.g., PDGF, or platelet-derived growth factor and VEGF, or vascular endothelial growth factor), are degraded or inactivated by proteolytic cleavage, causing delayed wound recovery [1].

Systemically administered proteolytic enzymes, such as trypsin, chymotrypsin, and bromelain, along with bioflavonoids like rutin, can restore this balance by supporting the natural enzymatic processes required for wound healing [8]. High concentrations of proteolytic enzymes have been shown to decelerate the inflammatory cycle, enhance postoperative healing, and reduce pain and inflammation. These enzymes provide a safer and more comprehensive approach to minimizing post-surgical complications, with reduced risks of adverse events [9,10].

Exogenous trypsin exhibits anti-inflammatory and anti-infective properties by reducing vascular permeability, inhibiting C-reactive protein (CRP) elevation, and promoting tissue repair, making it beneficial for wound care [11,12].

Bromelain, a protease from pineapple, reduces inflammation by lowering bradykinin, inhibiting prostaglandin (PGE2) formation, and promoting fibrinolysis for edema reabsorption [13,14]. It aids wound care by removing eschar without harming healthy and viable tissues, facilitating clean dermal and subdermal tissue, and promoting wound closure [15].

Rutoside, a bioflavonoid, offers antioxidant, anti-inflammatory, and vasoprotective properties. It stimulates wound healing, augments the tensile strength of scar tissue, and inhibits pro-inflammatory factors, such as thromboxane A2 (TXA2), thereby reducing platelet aggregation and attenuation of pro-inflammatory factors [16-18].

Gastrointestinal disturbances are the most frequently reported adverse events with systemic enzyme therapy, highlighting the need for a comprehensive evaluation of its safety and effectiveness in real-world settings [19,20]. Therefore, this study aimed to assess the safety and efficacy of Tibrolin^®^ (fixed-dose combination (FDC) of trypsin, bromelain, and rutoside tablets) in improving wound healing and alleviating acute pain in patients following uncontaminated surgeries.

## Materials and methods

Study design and setting

It was an open-label, prospective, multi-center, randomized, controlled, comparative, phase-IV clinical study conducted at four geographically distributed sites across India.

This study was carried out in accordance with the protocol and the requirements of New Drugs and Clinical Trials Rules, 2019; Ethical Guidelines for Biomedical Research on Human Participants, Indian Council of Medical Research, 2017; International Council for Harmonization Guidelines E6 (R2) for Good Clinical Practice; Declaration of Helsinki (World Medical Association); and Ethical Principles for Medical Research Involving Human Subjects (Brazil, October 2013).

The study was initiated after receiving approval from the Drug Controller General of India (DCGI) and the respective institutional ethics committees at each of the study centers. The trial was registered with the Clinical Trial Registry of India (CTRI No. CTRI/2021/035082).

Eligibility criteria

The study enrolled 200 patients of both sexes, aged between 18 and 65 years, who underwent elective, clean, and uncontaminated minor orthopedic surgery. All patients provided written informed consent to participate in the study.

Patients were excluded from this study if they had a history of hypersensitivity to any of the ingredients of the formulation, had hepatocellular insufficiency, hepatic failure, or active liver disease, had severe renal impairment, had a hereditary coagulation disorder, or were pregnant or breastfeeding.

Treatments

Eligible patients were randomly assigned (1:1) to Tibrolin^®^ (FDC of trypsin 48 mg, bromelain 90 mg, and rutoside 100 mg tablets) marketed by Zuventus Healthcare Limited, Mumbai, India, or Chymoral Forte^®^ (trypsin-chymotrypsin tablets with 100,000 armor units of enzymatic activity) marketed by Torrent Pharmaceuticals Ltd, Ahmedabad, India. The medications were administered as two tablets orally thrice a day post-operatively for seven days, approximately at the same time each day, as per the treatment schedule.

Study procedure

All the patients with a duly signed informed consent form, who fulfilled the inclusion criteria, were enrolled in the study. The study treatment was assigned as per the randomization schedule. The treatment lasted for seven days and required four visits, which included the screening check/baseline, on days 3, 5, and 7. The patient’s vital signs (blood pressure, heart rate, temperature, and respiration rate) were monitored during the treatment period, and adverse events were recorded on each follow-up visit for safety evaluation. Serum creatinine, BUN, total bilirubin, serum glutamic-oxaloacetic transaminase (SGOT), serum glutamic-pyruvic transaminase (SGPT), complete blood count (CBC), erythrocyte sedimentation rate (ESR), and CRP were checked at baseline and on day 7. Using the patient's clinical signs and symptoms, the improvement in wound healing and intensity of pain were also assessed by the orthopedic surgeons on days 3, 5, and 7. At the end of the study, the global assessment of treatment tolerability was evaluated based on the patient’s and the investigator’s response to the treatment. All the recruited patients received a patient diary with information on the seven-day medicine administration regimen in order to monitor treatment compliance. The patient's treatment compliance was evaluated at each follow-up session through the questionnaire and patient diary.

Study assessment

Primary Outcome: Safety Assessment

The primary endpoint of the present study was the percentage of patients reporting incidences of adverse events. Safety was assessed throughout the study based on observed adverse events and their relation to the investigational drug ascertained according to the WHO-Uppsala Monitoring Center scale.

Secondary Outcome: Efficacy Assessment

The secondary endpoints of the study included assessing the mean change in individual and total scores of surgical wound symptoms, as well as the mean change in Numerical Pain Rating Scale (NPRS) scores from baseline to day 7. Improvement in wound healing was measured by evaluating the severity of symptoms, such as erythema, edema, discharge, induration, local irritation, and tenderness. These symptoms were assessed on days 3, 5, and 7 of the study using a four-point Likert scale (0 = absent, 1 = mild, 2 = moderate, and 3 = severe). The mean of all wound symptom scores at baseline and at the end of the study was calculated and represented as the total wound symptom score. Pain intensity was evaluated using a validated NPRS scale, an 11-point Likert scale (0 = no pain and 10 = worst possible pain) [21]. The change in pain intensity from baseline and at each follow-up visit was recorded.

Also, the investigators and patients provided the global assessment of treatment tolerability using a Likert-type scale reading from 1 to 5, indicating (1 = excellent, 2 = good, 3 = average, 4 = no response, and 5 = poor) on day 7 for each patient.

Statistical analysis

A sample size of 174 subjects was needed to assess the study objective. Considering the 10% dropout, a total sample size of 200 subjects was planned to be enrolled in this study. Baseline characteristics of the enrolled patients were represented as mean ± standard deviation (SD) or percentage (%).

Safety Analysis

Safety was assessed by evaluating the total number of patients reporting side effects. Any adverse event that occurred was measured as the percentage of side effects reported by the patients.

Efficacy Analysis

All the individual and total scores of surgical wound symptoms were represented as mean ± SD. The total score of surgical wound symptoms was calculated as the average of all individual wound scores reported at each visit. The mean change in the individual wound symptom score from baseline was calculated as the mean difference in wound symptom scores at each visit from baseline. Similarly, the mean change in the total wound symptom score from baseline was calculated as the mean difference in wound symptom scores at each follow-up visit from baseline. To account for the mean change in NPRS score from baseline, the mean difference in pain score at each follow-up visit from baseline was calculated. The global assessment of treatment tolerability, based on the investigators' and patients' responses to treatment, was calculated as a percentage of each response.

Using two-sample unpaired t-tests, the two treatment groups were compared for the decrease in aggregate score and pain score. Using a paired t-test, the decrease in wound symptoms and pain scores was compared within groups. Additionally, a subgroup analysis was also conducted. The statistical significance was set at p < 0.05.

## Results

Study patients

**Figure 1:**
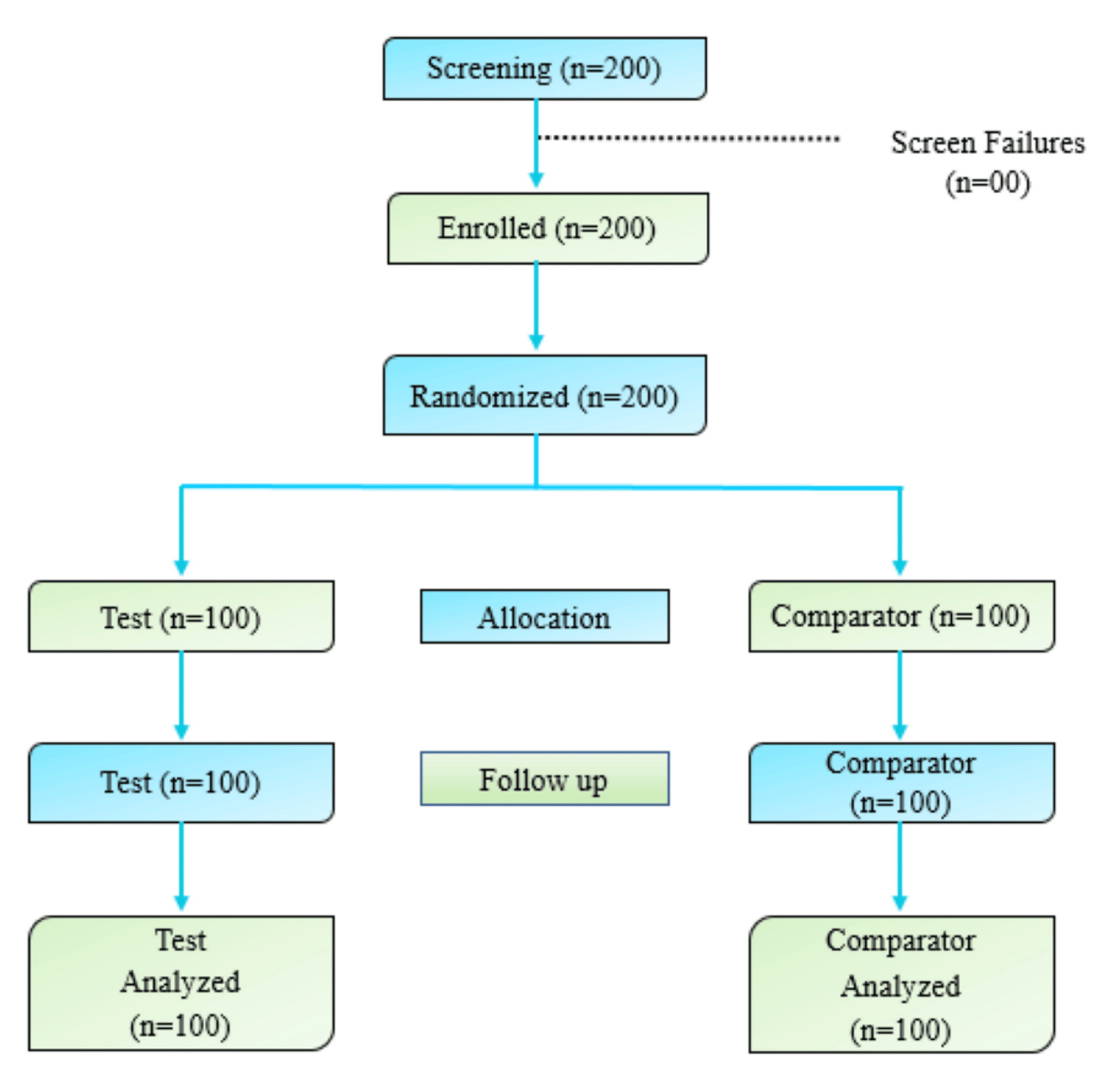
Disposition of patients

A total of 200 eligible patients undergoing minor surgeries, including fractures, dislocations, soft tissue injuries, abscesses, excisions, tendon repairs, arthroscopies, and implant removals, were enrolled in the study. They were randomized in a 1:1 ratio to receive either Tibrolin^®^ or Chymoral Forte^®^ during the trial, which was conducted from August 2021 to December 2021. The mean age of the enrolled patients was 35.88 ± 12.06 years. None of the patients dropped out or discontinued the study. The most commonly consumed concomitant medications in both groups were antibiotics, vitamins and minerals, and gastrointestinal/acid suppressants/prokinetics after surgery. Overall, the demographic and baseline characteristics were comparable between the treatment groups. The CONSORT flow diagram of the study is represented in Figure 1. The detailed demographic data are presented in Table 1.

**Table 1:** Patient demographic data and baseline characteristics ^ represents n (%) (analyzed using the Chi-square test); # represents mean ± SD (analyzed using the unpaired t-test).

Parameter	Test	Comparator	p-value
Gender ^			0.631
Male	75 (75.00%)	72 (72.00%)	-
Female	25 (25.00%)	28 (28.00%)	
Age (years)^#^	36.53 ± 11.94	35.23 ± 12.21	0.4381
Weight (kg)^ #^	63.67 ± 10.93	62.14 ± 9.64	0.2949
Height (cm)^ #^	165.20 ± 8.04	163.61 ± 7.59	0.1514
BMI (kg/m^2^)^ #^	23.19 ± 2.54	23.13 ± 2.55	0.877

Safety assessment

Incidences of Adverse Events Observed

There were no clinically significant changes observed in the vital signs of the patients between each visit. No adverse events were observed during the study. Furthermore, no severe or treatment-related adverse events were experienced by the patients.

Efficacy assessment

Reduction in the Severity of Individual Wound Symptoms

The reduction in wound symptom scores for erythema, edema, wound discharge, induration, local irritation, and tenderness on each follow-up visit, when compared to the baseline visit, was significantly high (p < 0.001) in the test group. A similar reduction was seen in the comparator group. A significant resolution in discharge symptoms (1.07 ± 1.15 on day 0 to 0.02 ± 0.14 on day 7) for the test group indicates improved drainage of wound fluid, suggesting improved wound healing. Additionally, a significant reduction (p = 0.035) in induration symptoms was seen from postoperative day 3 between the two groups, indicating a probability of improved effect on wound healing with the use of systemic oral enzymes.

Reduction in the Severity of Total Wound Symptoms

The total wound symptom scores significantly decreased in both the test group (10.66 ± 3.40 to 1.66 ± 2.01) and the comparator group (10.88 ± 3.17 to 1.56 ± 1.91) by day 7 (p < 0.001), with no significant difference between groups (p = 0.554). The observed scores for each outcome have been tabulated in Table 2.

**Table 2:** Mean change in wound symptom score ^a ^Day 0 versus Days 3, 5, and 7 mean differences between groups from Day 0 to Days 3, 5, and 7 (analyzed using the unpaired t-test). Note: p < 0.001, change in mean symptom score within the group from Day 0 to Days 3, 5, and 7 (analyzed using the paired t-test).

	Test (mean ± SD)	Comparator (mean ± SD)	Between-group difference		
			Mean difference (95% CI) ^a^	p-value	
Erythema					
Day 0	2.21 ± 0.70	2.17 ± 0.71	-	-	
Day 3	1.74 ± 0.60	1.74 ± 0.60	-0.04 (-0.18; 0.10)	0.572	
Day 5	1.03 ± 0.61	1.04 ± 0.51	-0.05 (-0.23; 0.13)	0.579	
Day 7	0.31 ± 0.46	0.28 ± 0.45	-0.01 (-0.22; 0.20)	0.926	
Edema					
Day 0	2.06 ± 0.79	2.08 ± 0.73	-	-	
Day 3	1.70 ± 0.76	1.68 ± 0.60	0.04 (-0.09; 0.18)	0.562	
Day 5	1.07 ± 0.62	1.02 ± 0.59	0.07 (-0.12; 0.26)	0.47	
Day 7	0.41 ± 0.53	0.39 ± 0.53	0.04 (-0.20; 0.28)	0.741	
Discharge symptom					
Day 0	1.07 ± 1.15	1.15 ± 1.17	-	-	
Day 3	0.69 ± 0.84	0.78 ± 0.85	-0.01 (-0.15; -0.13)	0.889	
Day 5	0.36 ± 0.56	0.34 ± 0.48	-0.10 (-0.14; 0.34)	0.411	
Day 7	0.02 ± 0.14	0.00 ± 0.00	-0.10 (-0.22; 0.42)	0.541	
Induration symptoms					
Day 0	1.70 ± 0.83	1.89 ± 0.71	-	-	
Day 3	1.23 ± 0.81	1.26 ± 0.66	0.16 (0.01; 0.31)	0.035	
Day 5	0.65 ± 0.72	0.59 ± 0.62	0.25 (0.06; 0.44)	0.011	
Day 7	0.26 ± 0.48	0.24 ± 0.43	0.21 (0.00; 0.42)	0.049	
Tenderness					
Day 0	1.88 ± 0.74	1.77 ± 0.65	-	-	
Day 3	1.26 ± 0.86	1.16 ± 0.80	-0.01 (-0.19; 0.17)	0.912	
Day 5	0.76 ± 0.75	0.68 ± 0.69	-0.03 (-0.22; 0.16)	0.754	
Day 7	0.37 ± 0.53	0.36 ± 0.50	-0.10 (-0.30; 0.10)	0.319	
Local irritation					
Day 0	1.74 ± 0.76	1.82 ± 0.70	-	-	
Day 3	1.15 ± 0.85	1.20 ± 0.74	0.03 (-0.13; 0.19)	0.704	
Day 5	0.66 ± 0.78	0.63 ± 0.66	0.11 (-0.09; 0.31)	0.282	
Day 7	0.29 ± 0.46	0.29 ± 0.48	0.08 (-0.12; 0.28)	0.435	
Total wound symptom					
Day 0	10.66 ± 3.40	10.88 ± 3.17	-	-	
Day 3	7.77 ± 3.12	7.82 ± 2.51	0.17 (-0.34; 0.68)	0.515	
Day 5	4.53 ± 2.73	4.30 ± 2.25	0.45 (-0.39; 1.29)	0.293	
Day 7	1.66 ± 2.01	1.56 ± 1.91	0.32 (-0.74; 1.38)	0.554	

Reduction in Pain Intensity Score

In the test group, pain intensity decreased significantly from 6.43 ± 1.68 to 0.85 ± 0.85 (p < 0.001), and in the comparator group, from 6.42 ± 1.69 to 0.93 ± 0.90 (p < 0.001) by day 7, with no significant difference in mean change between groups (p = 0.737). The results are summarized in Table 3.

**Table 3:** Mean change in pain intensity score by Numerical Pain Rating Scale (NPRS) score Data is expressed as mean ± SD. ^a^ Day 0 versus Days 3, 5, and 7 in both groups (analyzed with the unpaired t-test); ^b^ Analyzed with the unpaired t-test (p < 0.001 as compared to Day 0).

	Test	Comparator	Between-group difference	
			Mean difference (95% CI) ^a^	p-value
Day 0	6.43 ± 1.68	6.42 ± 1.69	-	-
Day 3	4.45 ± 1.18 ^b^	4.59 ± 1.12 ^b^	-0.15 (0.43; 0.13)	0.300
Day 5	2.60 ± 1.01 ^b^	2.54 ± 0.98 ^b^	0.05 (-0.39; 0.49)	0.823
Day 7	0.85 ± 0.85 ^b^	0.93 ± 0.90 ^b^	-0.09 (-0.62; 0.44)	0.737

Global assessment of patient and investigator’s response to therapy on day 7

**Figure 2:**
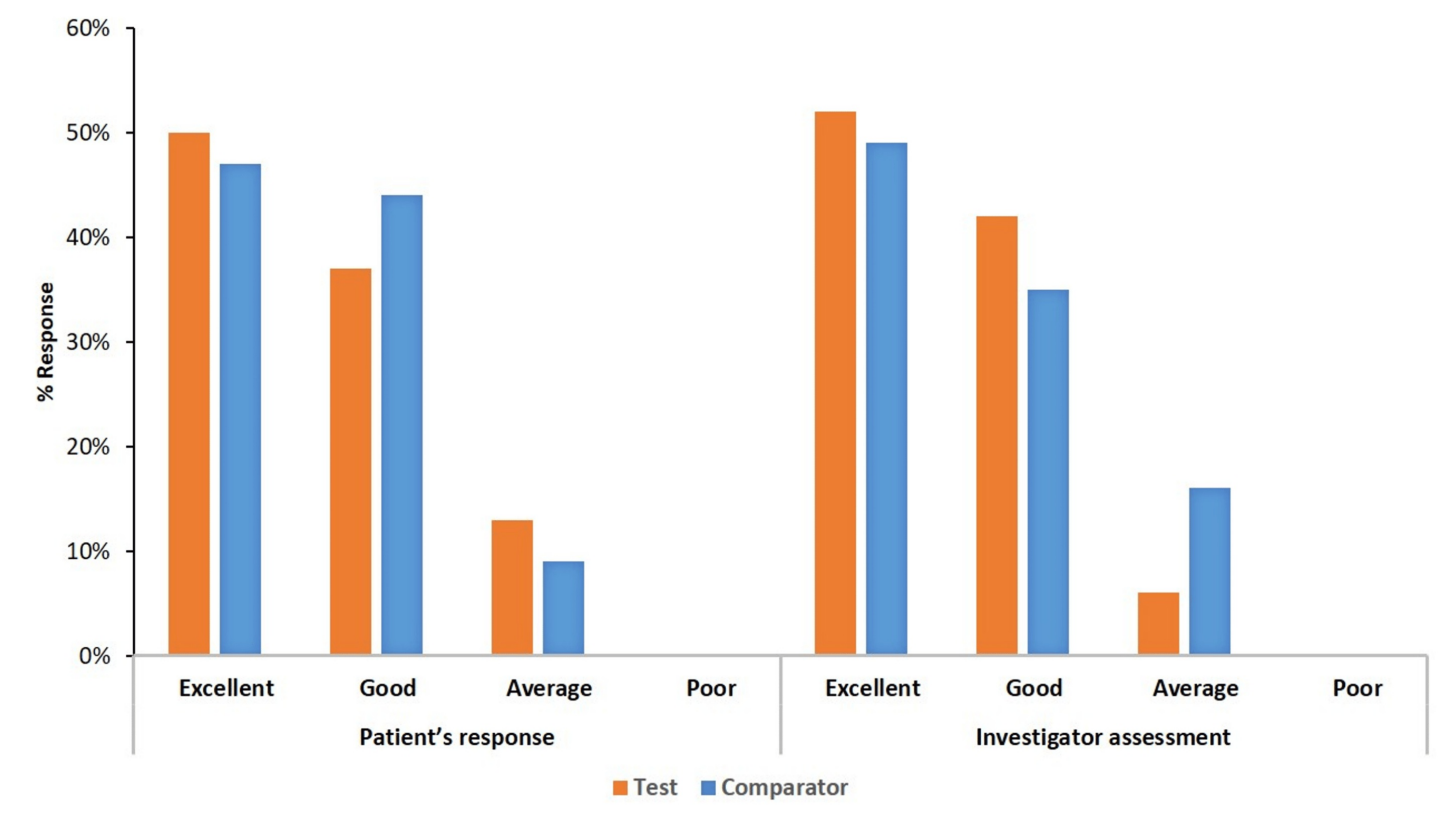
Global assessment of treatment tolerability

There were no reports of dose adjustments or study modifications brought on by intolerance or adverse effects related to the study drug. About 50% of patients and clinical investigators rated the study medication as excellent, while more than 35% of the remaining patients and clinical investigators recorded a good response to the study medication. The details of the responses recorded have been graphically represented (Figure 2).

## Discussion

Proteases have emerged as notable biochemical agents in wound healing [22]. Specifically, systemic enzyme therapy with a trypsin-bromelain-rutoside combination has long been used to counter various wound-associated inflammatory conditions. These enzymes are gaining attention as a promising adjunct in wound management due to their ability to facilitate wound healing, restore protease balance, and enhance healing outcomes - particularly in non-healing wounds where endogenous mechanisms are insufficient [23]. The efficacy and safety of oral proteolytic enzymes for decreasing wound symptoms and swelling post-surgery, as well as in traumatic conditions, have been evaluated in previous clinical trials [24,25].

In this study, we observed a highly significant pain reduction (p < 0.001) in both treatment groups. There was an 87% reduction in pain score by the end of the study period in the test group and 86% in the comparator group, indicating that the test treatment was equally efficacious compared to the comparator. A similar study conducted by Ueberall et al. found a 41% reduction in pain intensity, both at rest and during movement, in groups treated with the FDC of bromelain, trypsin, and rutoside, as well as in the diclofenac group (p < 0.001). This finding confirms that bromelain and other proteolytic enzymes are an effective alternative to non-steroidal anti-inflammatory drugs (NSAIDs) in reducing postoperative pain [26].

Dhadiwal et al. demonstrated that the oral administration of a trypsin-bromelain-rutoside combination resulted in about a 58% reduction in pain and swelling associated with diverse orthopedic conditions. This significant reduction in pain (p < 0.001) ascertains the anti-inflammatory and analgesic activity of oral proteolytic enzymes [27].

Each follow-up visit after initiation of wound treatment revealed a significant decrease (p < 0.001) in the total wound symptom score for both groups. There was about an 85% improvement in the total wound symptom score by the end of the study period in both treatment groups, indicating Tibrolin^®^ to be as efficacious as the trypsin-chymotrypsin combination. Additionally, the test group exhibited no wound discharge by the end of the treatment schedule and a more pronounced improvement in induration symptoms (p = 0.049), along with a significant difference between and within the treatment groups. These results indicate progressive wound healing.

A study by Mungantiwar et al. reported the safety and effectiveness of oral bromelain, trypsin, and rutoside trihydrate enzyme combination therapy over serratiopeptidases for the treatment of minor surgical wounds, claiming enzyme combination therapy to be equally safe and more efficacious as compared to serratiopeptidase in wound healing [28].

In another study by Brown et al., who studied the effect of proteolytic enzymes on skin wound healing, they reported accelerated soft-tissue wound healing in 77% of normal, healthy subjects studied. The 17% acceleration of wound-healing time was significant (p < 0.001). In subjects responding to oral proteolytic enzyme supplements, less redness in the wounds was observed, which may have been associated with less inflammation [9].

Thus, in accordance with the current literature and observations from similar trials, Tibrolin^®^ efficiently improves clinical symptoms in patients with postoperative wound symptoms and pain (p < 0.001) within seven days of therapy. Also, considering the relatively better safety profile of these natural-origin drugs, this therapy provides a very promising alternative to conventional therapies for managing clean and uncontaminated minor orthopedic surgical wounds [29]. Tibrolin^®^ was well tolerated by all patients, and no significant adverse events were observed throughout the study period.

## Conclusions

The concept of wound management has eventually evolved to provide a systematic approach to removing the barriers to natural healing and enhancing the effects of proteolytic therapies, to overcome the trapping of wounds in a constant inflammatory state, which fails to progress through the normal healing stages.

We observed that Tibrolin^®^ significantly improved postoperative wound symptom scores and pain. Administration of Tibrolin^®^ efficiently relieves the symptoms associated with wound/surgical procedures, along with additional analgesic effects. These advantages justify the inclusion of Tibrolin^®^ as a part of empirical therapy in the management of elective clean and uncontaminated minor orthopedic surgical wounds.
